# Comparison of Coenzyme Q10 (Ubiquinone) and Reduced Coenzyme Q10 (Ubiquinol) as Supplement to Prevent Cardiovascular Disease and Reduce Cardiovascular Mortality

**DOI:** 10.1007/s11886-023-01992-6

**Published:** 2023-11-16

**Authors:** Johannes-Paul Fladerer, Selina Grollitsch

**Affiliations:** 1 Institute of Pharmaceutical Sciences, University of Graz, Beethovenstraße 8, 8010 Graz, Austria; 2 Apomedica Pharmazeutische Produkte GmbH, Roseggerkai 3, 8010 Graz, Austria

**Keywords:** Coenzyme Q10, Heart failure, Ubiquinone, Ubiquinol

## Abstract

**Purpose of Review:**

According to the World Health Organization (WHO), cardiovascular disease is the leading cause of death worldwide. Heart failure has been defined as a global pandemic leading to millions of deaths. Recent research clearly approved the beneficial effect of Coenzyme Q10 supplementation in treatment and prevention of cardiovascular disease in patients with heart failure in clinical trials but did not distinguish between the oxidised form CoQ10 and reduced form CoQH2 of Coenzyme Q10. The aim of this study is to determine differences in medical application of CoQ10 and CoQH2 supplementation and evaluate the efficacy of CoQ10 and CoQH2 supplementation to prevent cardiovascular disease in patients with heart failure.

**Recent Findings:**

A PubMed search for the terms “ubiquinone” and “ubiquinol” was conducted, and 28 clinical trials were included. Our findings go along with the biochemical description of CoQ10 and CoQH2, recording cardiovascular benefits for CoQ10 and antioxidative and anti-inflammatory properties for CoQH2. Our main outcomes are the following: (I) CoQ10 supplementation reduced cardiovascular death in patients with heart failure. This is not reported for CoQH2. (II) Test concentrations leading to cardiovascular benefits are much lower in CoQ10 studies than in CoQH2 studies. (III) Positive long-term effects reducing cardiovascular mortality are only observed in CoQ10 studies.

**Summary:**

Based on the existing literature, the authors recommend CoQ10 instead of CoQH2 to treat and prevent cardiovascular disease in patients with heart failure.

## Introduction

According to the World Health Organization (WHO), cardiovascular disease (CVD) is the leading cause of death worldwide [1]. Heart failure (HF) has been defined as a global pandemic leading to millions of deaths [2]. HF is described as clinical syndrome with symptoms and/or signs caused by a structural and/or functional cardiac abnormality and corroborated by elevated natriuretic peptide levels and/or objective evidence of pulmonary or systemic congestion [3]. During the last decades, Coenzyme Q_10_ has been established as adjunctive therapy for CVD and HF [4].

**Fig. 1:**
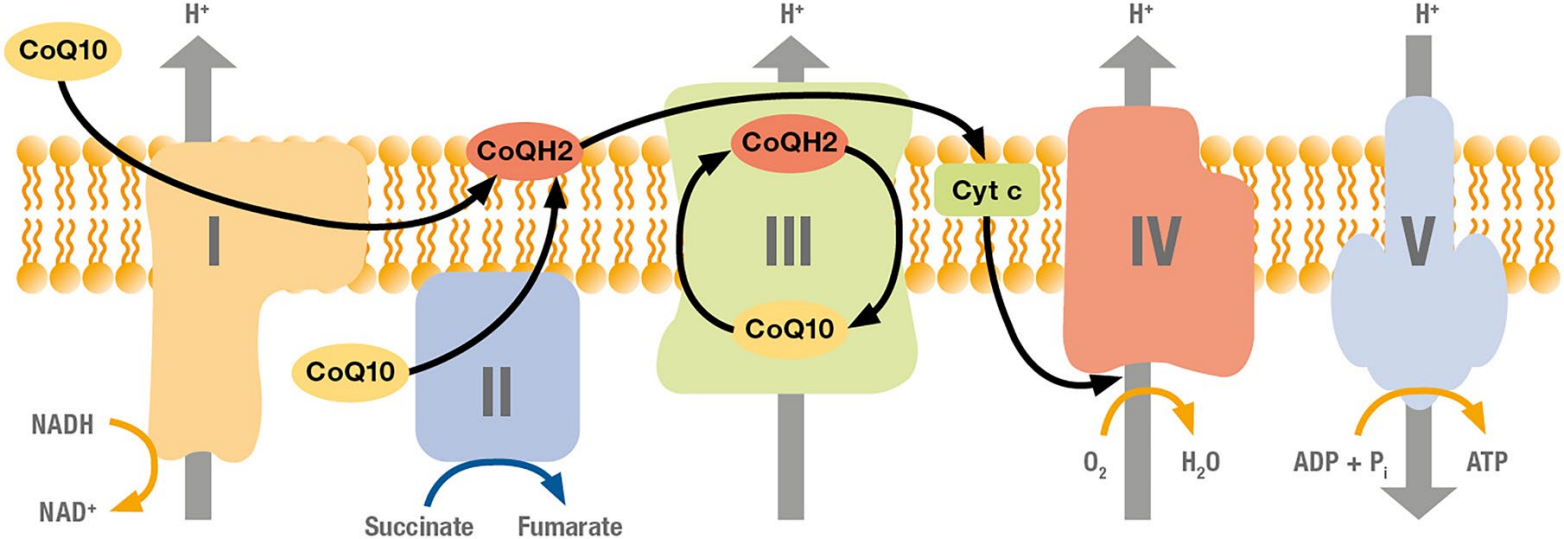
Function of CoQ10 in the mitochondrial respiratory chain. CoQ10 is an essential transfer molecule for complex I and complex II at the beginning of the respiratory chain

Coenzyme Q_10_ is a redox molecule occurring in the human body in 2 bioactive states, ubiquinone (CoQ10) as oxidised state and ubiquinol (CoQH2) as reduced state [5]. Both redox forms of Coenzyme Q_10_ are bioactive and important for human health [6]. CoQ10 is essential for cellular adenosine phosphate (ATP) energy production [7] as it shuttles electrons from complexes I and II to complex III of the mitochondrial respiratory chain (Fig. 1). CoQH2 is an important lipid-soluble antioxidant preventing peroxidation of the low-density lipoproteins in the blood circulation [8] with additional anti-inflammatory activity [9].

A slightly better water solubility and a lack of understanding absorption and transfer of CoQ10 and CoQH2 have led to misleading interpretations pushing CoQH2 as more bioactive form [10•]. Therefore, it is important to notice that (I) CoQH2 is very unstable [11] and under normal conditions oxidised to CoQ10 [12], (II) CoQH2 has to be oxidised to CoQ10 before it can be absorbed in enterocytes [6], (III) the bioavailability of CoQ10 and CoQH2 mainly depends on crystal dispersion status and carrier oil composition [13].

Interestingly, only CoQ10 is synthesised in the human body by way of the mevalonate pathway, an essential metabolic pathway including byproducts like cholesterol and other isoprenoids. And this shared dependence on the mevalonate pathway was the reason for focusing attention to CoQ_10_ in CVD [14].

Recent reviews clearly approved the beneficial effect of Coenzyme Q10 supplementation in treatment and prevention of CVD in clinical trials but did not distinguish between CoQ10 and CoQH2 supplementation [15]. As comparative clinical trials are missing and misleading marketing claims are revealed [10•], a critical view comparing the effectivity of supplementation with CoQ10 and CoQH2 in CVD therapies is needed.

## Objectives

Our goal is to determine differences in medical application of CoQ10 and CoQH2 supplementation and evaluate the efficacy of CoQ10 and CoQH2 supplementation to prevent cardiovascular diseases. The final aim is to identify the most promising supplement to reduce cardiovascular mortality in patients with heart failure. We focused on the medical evidence that can be found in the literature.

## Methods

### Search Methods

We searched PubMed for the term “ubiquinone” and “ubiquinol” from 5 to 12th July 2023. We applied no language restrictions. We used the filter “randomized controlled trial” and searched for results published between 1993 and 2023.

### Selection Criteria

Only randomised, double blind, placebo-controlled, parallel or cross‐over trial has been included. Trials investigated the supplementation of CoQ10 or CoQH2 alone or with one other single supplement. We excluded any trials involving multifactorial lifestyle interventions to avoid confounding. Trials including children or lacking necessary information or comparability have been excluded from the evaluation of ubiquinone and ubiquinol supplementation to prevent cardiovascular diseases. We excluded studies with a daily CoQ10 or CoQH2 intake lower than 50 mg. Follow-up-studies with own objectives are treated as separate studies.

### Types of Outcome Measures

To determine differences in medical application of CoQ10 and CoQH2 supplementation, we assigned the studies to the following applications: antioxidative activity, bronchial diseases, cancer, cardiovascular diseases, eye diseases, hepatic diseases, Huntington, infections, infertility, inflammation, mental health, metabolic syndrome, migraine, mitochondrial dysfunction, pain, Parkinson, physical health, polycystic ovary syndrome, pregnancy, presbycusis, statin associated pain, others.

In case of the evaluation of CoQ10 and CoQH2 as prevention of cardiovascular disease, we recorded reduced cardiovascular mortality, clinical status, echocardiography, 6-min walk test, NYHA, E/e´, EF, B-type natriuretic peptides (BNP) and hospitalisation.

We further compared the reduction of cardiovascular mortality in patients with heart failure as main result to identify the most promising supplement.

### Data Collection and Analysis

Two authors independently selected trials for inclusion, abstracted data and assessed the risk of bias.

## Results

**Fig. 2:**
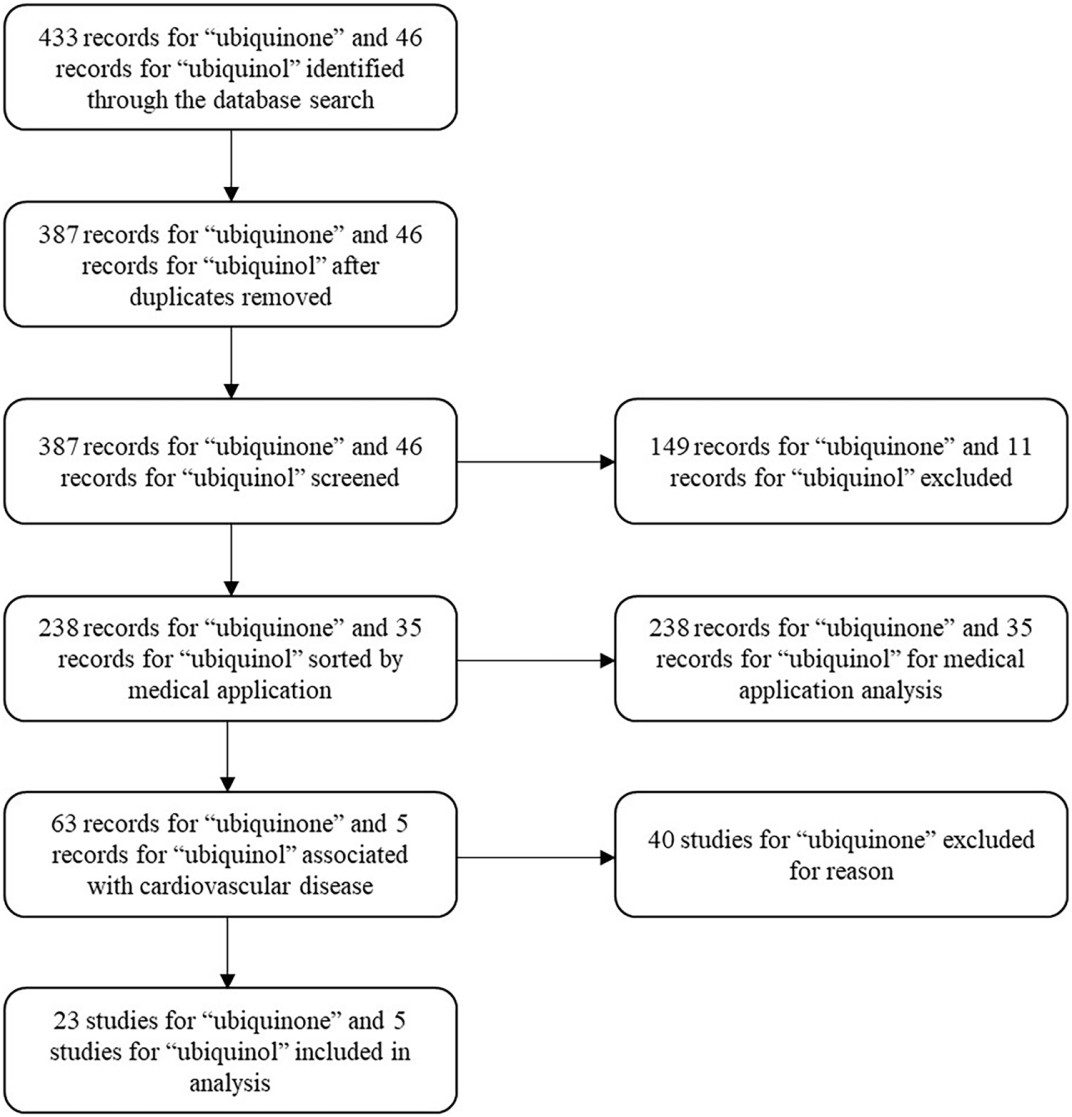
Study flow diagram

We identified 238 randomised controlled trials for ubiquinone and 35 for ubiquinol, which were sorted by medical application. Twenty-three studies of ubiquinone and 5 of ubiquinol were included to analyse their potential to prevent cardiovascular disease. These 28 studies were compared according to the ability of the given supplements to reduce cardiovascular mortality in patients with heart failure (Fig. 2).

### Different Medical Applications of CoQ10 and CoQH2 Supplementation

**Fig. 3:**
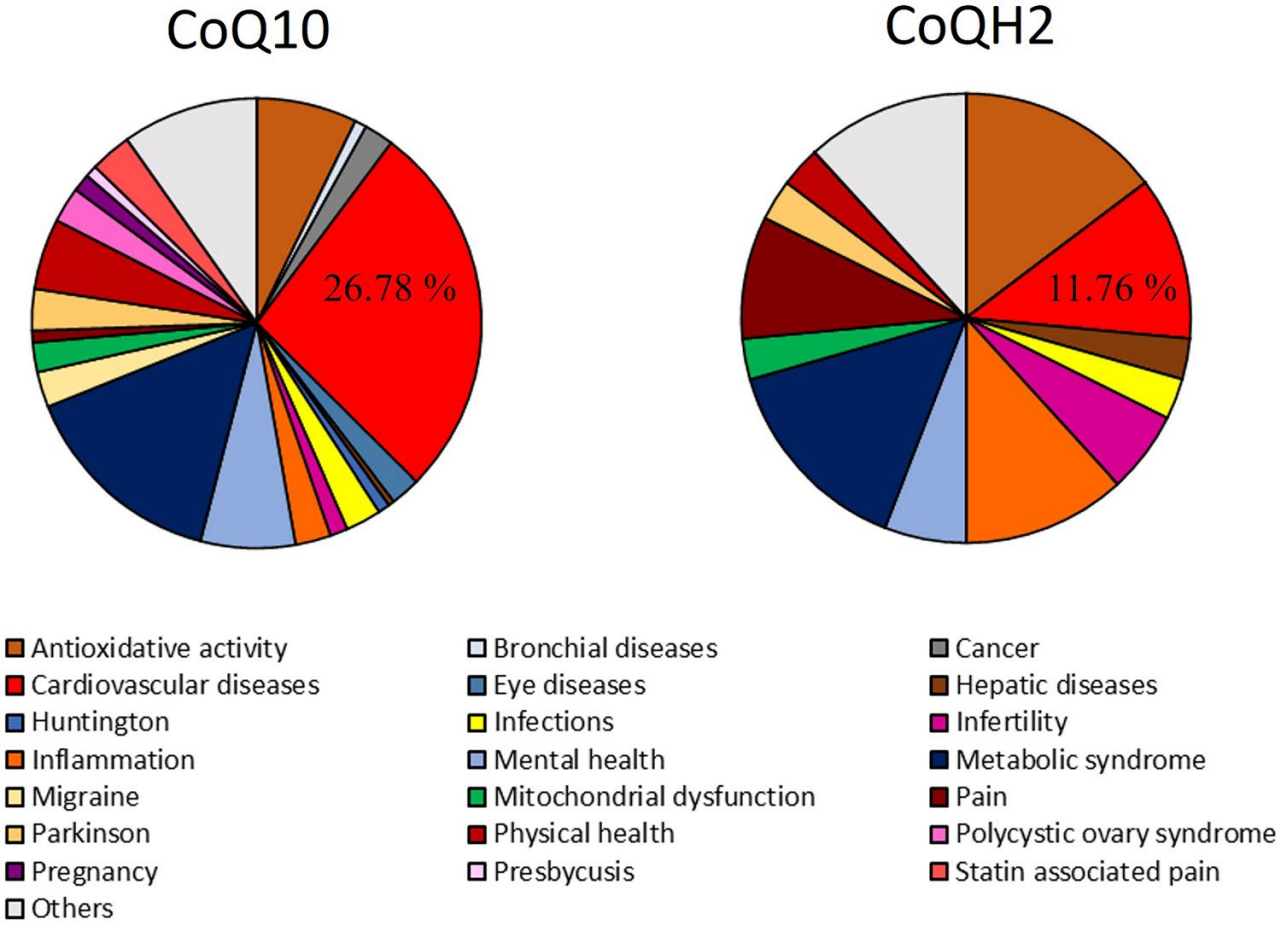
Comparison of CoQ10 and CoQH2 by application. Results are demonstrated as %. Cardiovascular diseases are marked in red

We associated a total of 273 studies with 21 medical applications. Studies with unclear applications or with applications recorded only once are included in the group “Others”. According to the different study numbers of CoQ10 and CoQH2, relative results are presented in Fig. 3.

**Fig. 4:**
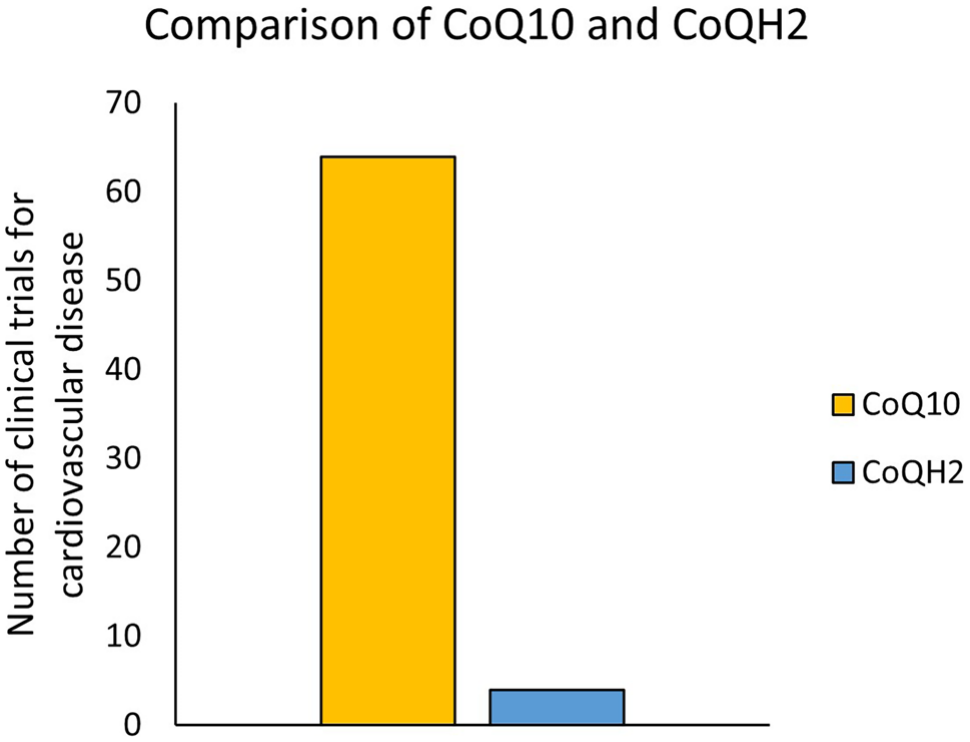
Comparison of CoQ10 and CoQH2. Given is the number of clinical trials associated with cardiovascular disease for both supplements

By comparison of applications, CoQ10 can be identified as promising agent to treat cardiovascular diseases while CoQH2 can be preferred for treatment of inflammation and antioxidative activity. These findings go along with the biochemical description of CoQ10 and CoQH2 [7, 8]. The different clinical evidence for the usage of CoQ10 and CoQH2 as supplement for cardiovascular disease is demonstrated in Fig. 4.

At this point, it should be mentioned that the enormous difference in the number of conducted studies (6.8-fold difference) suggests that CoQ10 is better investigated than CoQH2. The number of clinical trials for CoQH2 is limited [10•].

### Effects of CoQ10 and CoQH2 on Cardiovascular Diseases

Results of 28 studies (23 original studies and 5 follow-up studies with specific aims) investigating the effects of CoQ10 and CoQH2 on cardiovascular diseases are demonstrated in Table 1. In the original studies, 3523 are included.

**Table 1:** Effects of CoQ10 and CoQH2 on cardiovascular diseases. Empty cells are used if information is missing

	**Author**	**Year**	**Probands included**	**Duration supplementation/(study) [weeks]**	**Daily intake (mg/d)**	**Effect**	**Reduced cardiovascular mortality (%)**
**CoQ10**	Judy et al. [16]	1993	20	6/(6)	100	Improved echocardiogram and improved EF	
	Morisco et al. [17]	1993	641	52/(52)	160	Reduced time of hospitalisation	
	Chello et al. [18]	1994	40	1/(1)	150	No reduced cardiovascular mortality	0
	Morisco et al. [19]	1994	6	4/(8)	200	Improved echocardiogram Improved EF	
	Singh et al. [20]	1998	144	4/(4)	120	Reduced cardiovascular mortality	15.90
	Berman et al. [21]	2004	32	12/(12)	60	Improved clinical status, improved 6-min walk test and improved NYHA class	
	Chew et al. [22]	2008	74	24/(24)	160	No improved E/e´ ratio	
	Makhija et al. [23]	2008	30	7/(7)	165	Reduced time of hospitalisation	
	Kuimov et al. [24]	2013		8/(8)	60	Improved clinical status	
	Mortensen et al. [25••]	2014	420	16/(104)	300	Improved NYHA class, reduced time of hospitalisation and reduced cardiovascular mortality	18.00
	Aslanabadi et al. [26]	2016	100	0.14/(0.14)	300	Reduced cardiovascular mortality	2.00
	Mortensen et al. [27]	2019	231	12/(96)	300	Improved NYHA class, improved EF, reduced time of hospitalisation and reduced cardiovascular mortality	11.00
	Sobirin et al. [28]	2019	28	4/(4)	300	Improved E/e´ ratio	
	Khan et al. [29]	2020	123	0.14/(0.14)	400	Reduced BNPs and reduced time of hospitalisation	
**CoQ10 & Selenium**	Kuklinski et al. [30]	1994	61	52/(52)	100	Reduced cardiovascular mortality	20.00
	Alehagen et al. [31••]	2013	443	260/(520)	200	Improved echocardiogram, improved EF, reduced BNPs and reduced cardiovascular mortality	6.70
	Alehagen et al. [32]	2015	443	260/(520)	200	Reduced cardiovascular mortality	17.90
	Alehagen et al. [33]	2015	443	148/(270)	200	Reduced cardiovascular mortality	3.50
	Alehagen et al. [34]	2016	668	208/(478.4)	200	Reduced cardiovascular mortality	8.00
	Alehagen et al. [35]	2018	434	208/(832)	200	Reduced cardiovascular mortality	10.60
	Alehagen et al. [36]	2021	213	208/(254)	200	Reduced cardiovascular mortality	10.00
	Opstad et al. [37]	2022	118	42/(520)	200	Reduced cardiovascular mortality	6.00
	Alehagen et al. [38]	2022	443	148/(148)	200	Improved echocardiogram, improved EF, reduced BNPs and reduced cardiovascular mortality	6.70
**CoQH2**	Orlando et al. [39]	2020	50	2/(26)	400	Improved echocardiogram, improved EF, no reduced cardiovascular mortality	0
	Kawashima et al. [40]	2020	14	12/(12)	400	No improved echocardiogram and improved endothelial function	
	Holmberg et al. [41]	2021	43	1/(1)	600	Improved EF and no reduced cardiovascular mortality	0
	Samuel et al. [42]	2022	39	12/(12)	300	No Improved E/e´ratio and no reduced BNPs	
**CoQH2 & Ribose**	Pierce et al. [43•]	2022	216	12/(12)	600	Improved clinical status, improved EF, reduced BNPs and no reduced cardiovascular mortality	0

Summarising results of Table 1 leads to three major outcomes: (I) the number of studies investigating the effect of CoQH2 on cardiovascular diseases is very limited; (II) In contrast to CoQ10, no CoQH2 study could clearly demonstrate a reduced cardiovascular mortality; (III) the used concentrations are much higher in studies investigating CoQH2.

According to these results, we conclude that based on the medical data available, CoQ10 is the more promising supplement to prevent cardiovascular diseases and to treat patients with heart failure. Further arguments for CoQ10 are the additive effect in combination with selenium [30, 31••] and the reduction of adverse effects of statin therapy by supplementation with CoQ10 [44–46]. Additionally, in all clinical trials included in this study, patients proceeded with their previous medication (statins, antihypertensives and others) and no interactions between CoQ10 and medicines could be observed.

At this point, it should be noticed that the Q-SYMBIO [25••] and KISEL-10 studies [31••] and their follow-up studies, all working on CoQ10, are the only clinical investigations clearly demonstrating a reduction in cardiovascular mortality in patients with heart failure over many years. Some studies working on CoQH2 are citing Q-SYMBIO and KISEL-10 studies to prove the cardiovascular effect of CoQH2 despite the fact that these studies are working on CoQ10 instead of CoQH2 [42]. Such misleading investigations are in detail discussed by Judy and Mantle [6, 10•].

## Discussion

Even 30 years ago, studies recorded the beneficial effect of CoQ10 supplementation alone or in combination with selenium to improve EF [16], reduce time of hospitalisation [17] and reduce cardiovascular mortality in patients with heart failure [30]. In contrast to that, clinical trial investigation on the effect of CoQH2 on patients with heart failure started much later in 2020 [39] while investigations concerning antioxidant and anti-inflammatory activity of CoQH2 have been conducted in 1993 [47, 48]. This difference in medical evidence makes it challenging to compare CoQ10 and CoQH2 supplementation according to its usage to treat patients with cardiovascular disease and heart failure. Additionally, most CoQH2 studies used much higher concentrations than CoQ10 studies. CoQ10 studies included in this research test concentrations between 60 and 300 mg/day with one exception using 400 mg/day [29]. Studies with CoQ10 with selenium additive test between 100 and 200 mg/day. In contrast to that, CoQH2 studies included test concentrations between 300 and 600 mg/day. The only CoQH2 study using 300 mg/day test concentration included only 39 patients, and no beneficial cardiovascular effects could be observed [42].

Taking a closer look at major studies including at least 200 patients reveal extreme differences between CoQ10 and CoQH2. The group of Morisco et al. [17] reported a significant reduction in hospitalisation time in a study including 641 patients with heart failure and CoQ10 supplementation of 160 mg/day. The most cited Q-SYMBIO study of Mortensen et al. [25••] included 420 patients with heart failure and applied 3 × 100 mg of CoQ10 per day. After 2 years, a significant reduced time of hospitalisation and an improved NYHA class could be recorded. And most important, the cardiovascular mortality decreased by 18% compared to placebo group. KISEL-10 studies [31••] included 443 patients and applied 200 mg of CoQ10 per day combined with 200 µg of selenium. These studies focused on long-term effects of CoQ10 on cardiovascular mortality in patients with heart failure. They recorded a decrease of cardiovascular mortality of 17.9% after 10 years [32] and of 10.6% after 12 years [35]. In contrast to that, the CoQH2 study of Pierce et al. [43•] included 216 patients and applied 600 mg of CoQH2 per day in combination with 15 g/day of ribose. They recorded improved clinical status, improved EF and reduced BNPs values, but no reduction of cardiovascular mortality. According to the higher concentration (600 mg/day instead of 200 or 300 mg/day) used in CoQH2 studies and the weaker recorded benefit for patients with heart failure, the usage of CoQ10 supplementation is recommended in therapies of heart failure and cardiovascular disease in general.

Interestingly, there are differences in clinical outcomes of CoQ10 and CoQH2 despite the fact that CoQ10 can be converted to CoQH2 and vice versa in the human body by at least five enzymes [6]. Possible reasons are a different stomach transit [49] and duodenal absorption [50]. At this point, it should be stated that more pharmacological studies are needed to provide a clear answer to this question. Mantle et al. consider that many different cell types may have the capacity to reduce CoQ10 to CoQH2 in the external cellular environment. And if this is found to be the case, then presumably any of the various cell types lining the gastrointestinal tract would be able to facilitate this conversion, and the requirement for supplemental CoQH2 to maximise absorption would be negated [6]. Finally, it has to be noted that clear differences in clinical outcomes occur between CoQ10 and CoQH2.

## Conclusion

As detailed above, our findings go along with the literature and the biochemical description of CoQ10 and CoQH2, recording cardiovascular benefits for CoQ10 and antioxidative and anti-inflammatory properties for CoQH2 [7, 8]. It has to be noted that there is a lack of clinical relevant trials and misleading marketing claims associating CoQH2 with cardiovascular benefits [10•]. Comparing the work of Morisco et al. [17] and Q-SYMBIO, KISEL-10 studies [25••, 31••] with the study of Pierce et al. [43•] lead to the following outcomes: (I) CoQ10 supplementation alone or in combination with selenium reduced cardiovascular death in patients with heart failure. This is not recorded for CoQH2. (II) Test concentrations leading to cardiovascular benefits are much lower in CoQ10 studies than in CoQH2 studies. (III) Positive long-term effects are only observed in CoQ10 studies. In these studies, reduced cardiovascular mortality is recorded even after 12 years. Based on the existing literature, the authors recommend CoQ10 instead of CoQH2 to treat and prevent cardiovascular disease in patients with heart failure.

## Data Availability

Data and additional informations are available on request.
